# RNA-Seq Analyses Identify Additivity as the Predominant Gene Expression Pattern in F1 Chicken Embryonic Brain and Liver

**DOI:** 10.3390/genes10010027

**Published:** 2019-01-07

**Authors:** Zhu Zhuo, Susan J. Lamont, Behnam Abasht

**Affiliations:** 1 Department of Animal and Food Sciences, University of Delaware, Newark, DE 19716, USA; zzhuo@udel.edu; 2 Department of Animal Science, Iowa State University, Ames, IA 50011, USA; sjlamont@iastate.edu

**Keywords:** gene expression patterns, heterosis, chicken, RNA-Seq

## Abstract

The superior performance of hybrids to parents, termed heterosis, has been widely utilized in animal and plant breeding programs, but the molecular mechanism underlying heterosis remains an enigma. RNA-Seq provides a novel way to investigate heterosis at the transcriptome-wide level, because gene expression functions as an intermediate phenotype that contributes to observable traits. Here we compared embryonic gene expression between chicken hybrids and their inbred parental lines to identify inheritance patterns of gene expression. Inbred Fayoumi and Leghorn were crossed reciprocally to obtain F1 fertile eggs. RNA-Seq was carried out using 24 brain and liver samples taken from day 12 embryos, and the differentially expressed (DE) genes were identified by pairwise comparison among the hybrids, parental lines, and mid-parent expression values. Our results indicated the expression levels of the majority of the genes in the F1 cross are not significantly different from the mid-parental values, suggesting additivity as the predominant gene expression pattern in the F1. The second and third prevalent gene expression patterns are dominance and over-dominance. Additionally, we found only 7–20% of the DE genes exhibit allele-specific expression in the F1, suggesting that *trans* regulation is the main driver for differential gene expression and thus contributes to heterosis effect in the F1 crosses.

## 1. Introduction

Crossbreeding for heterosis has tremendously improved agricultural production in recent centuries by taking advantage of non-additive genetic effects and producing progeny that exhibit greater qualities than both parents. Although heterosis has been intensively exploited by breeders to obtain desirable agronomic traits, laborious research effort is needed to identify varieties that result in useful heterosis when crossed. Identification of such plant or animal varieties may be facilitated by understanding the molecular basis of heterosis. Hypotheses of dominance [1,2] over-dominance [3,4] and epistasis [5,6] have long been proposed to explain the mechanistic bases of heterosis, which emphasize the effect of advantageous alleles, heterozygosity, and interaction of genes for multigenic traits, respectively. However, due to technological limitations, these hypotheses have remained overly unexamined up until the advent of genome-wide methods for polymorphisms and gene expression analysis.

Gene expression could be considered as an intermediate phenotype between genotypes and observable characteristics [7] and contributes to phenotypic heterosis [8]. Variation in gene expression, such as differential gene expression between parents and hybrids and allele-specific expression (ASE) in hybrids, may be key to understanding heterosis. Like an observable phenotype, the gene expression in the F1 may show an additive, dominance, over-dominance or under-dominance expression pattern. Acquiring such information through transcriptome-wide methods has recently gained traction in understanding gene expression basis of heterosis. Using microarray or RNA-Seq, heterosis has been analyzed in species such as rice [9], maize [10], soybeans [11], and silkworm [12]. However, to the best of our knowledge, there is no published report on gene expression analysis of heterosis in chickens or in any other livestock species.

In chickens, heterosis has been previously reported for growth, body composition, egg production and abdominal fatness [13,14,15]. Heterosis for economically important traits, such as mortality rate [16] and egg production [17], has been reported in F1 chickens produced from a cross between two genetically distant chicken lines, Fayoumi and Leghorn. We previously established reciprocal F1 crosses between highly inbred Fayoumi and Leghorn to study genomic imprinting and allele-specific expression using RNA-seq data from day 12 embryonic brain and liver samples of the parental lines and the reciprocal F1 crosses [18]. In the present study, we took advantage of these existing RNA-seq data and compared gene expression in F1 crosses and their prenatal lines. The aim of this paper is twofold. First, it examined heterosis effect in embryo weight at embryonic day 12. Second, it identified transgenerational gene expression patterns at an early stage of development to gain insight into gene expression heterosis.

## 2. Material and Methods

### 2.1. Ethical Statement

All animal protocols for production of the fertile eggs were conducted with the approval of Iowa State University IACUC Log #4-03-5425-G. No approval of University of Delaware AACUC was required for chicken embryo experiments.

### 2.2. Animals

**Figure 1:**
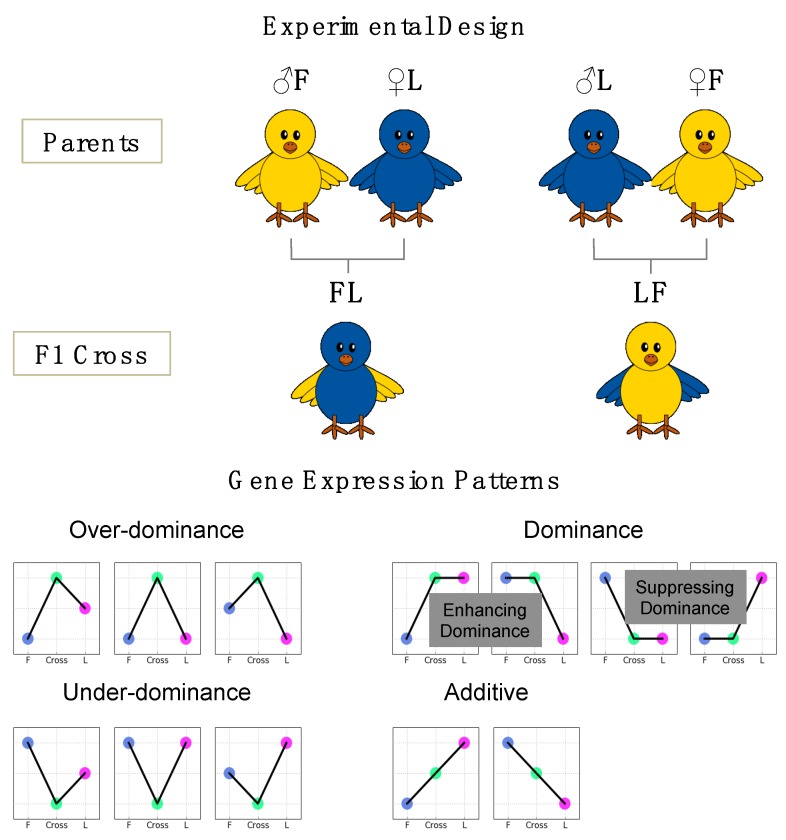
Experimental design and classification of gene expression patterns (F: Inbred Fayoumi line; L: Inbred Leghorn line; FL: Fayoumi × Leghorn cross; LF: Leghorn × Fayoumi cross; Cross: FL and LF).

Experimental design and sequencing strategy were described in detail previously [18]. Briefly, eggs of parental lines and their reciprocal crosses were obtained from two highly inbred chicken lines, Fayoumi and Leghorn, and incubated for 12 days (Figure 1). Fertility was checked at day 10 after incubation and infertile eggs were removed. Egg weights and embryo weights were recorded for 14 FL (Fayoumi × Leghorn cross), 4 LF (Leghorn × Fayoumi cross), 14 F (inbred Fayoumi line) and 16 L (inbred leghorn line) samples. Mid-parent values (MPVs) for egg weight and embryo weight were generated by averaging the values of randomly-paired F and L samples. Embryonic efficiency (percentage of embryo weight in egg weight) was evaluated [19]. Tissues sampled at day 12 were frozen immediately in liquid nitrogen and stored in −80 °C. The datasets of egg weight, embryo weight, and embryonic efficiency were not significantly deviated from normality (Shapiro-Wilks normality test) and were analyzed using ANOVA. All-pairs comparisons of means were performed using the Tukey-Kramer HSD test in JMP Pro 13.1.0 [20].

### 2.3. Analysis of Sequencing Data

Twenty-four samples (12 per tissue type) of male chicken embryos were chosen for further RNA-Seq study, which includes brain and liver samples from 4 FL, 4 LF, 2 F, and 2 L embryos. Total RNA was extracted from the selected samples, and cDNA libraries were prepared from polyadenylated RNA. A total of 1.5 billion 75-nucleotide reads were generated on HiSeq 2000 system (Illumina, San Diego, CA, USA), with an average of 64.8 million reads per sample. Raw sequencing reads are available in the NCBI Sequence Read Archive with accession number SRP102082. Reads were aligned to the Gallus gallus 5.0 genome assembly (Ensembl chicken release 89) using HISAT (v2.0.4) [21] with default parameters. Raw gene counts for each sample were obtained using Stringtie v1.3.0 [22]. Identification of new transcripts was disabled to facilitate comparison amongst samples. Differential expression of pairwise comparisons of F vs. L, FL vs. LF and Cross (including FL and LF) vs. each of F and L was analyzed using DESeq2 (v1.16.1) [23]. Additionally, a synthetic group of mid-parent gene expression values was calculated by taking the means of normalized gene count from combinations of paternal lines, and differentially expressed (DE) genes between the Cross and MPV groups were identified. An adjusted *p*-value less than 0.05 and a Log2 (fold change) (abbreviated as logFC) equal to or greater than 1 were applied to claim DE genes. The regularized log transformed counts (rLog(count)) from DESeq2 analysis were used to compare the relative abundance of gene expression between groups in the clustering analysis. Additionally, FPKM values for each sample were obtained using Cuffnorm (v2.2.1) [24] to normalize for library size and gene length.

### 2.4. Functional Analysis of Differentially Expressed Genes

A gene list for each tissue type was prepared by compiling all DE genes from pairwise differential expression analyses of F vs. L; Cross vs. each of F, L and MPV groups. The genes that were differentially expressed between the Cross and MPV groups were considered as genes showing a non-additive (dominance, over-dominance, and under-dominance) expression pattern (Figure 1). Over-dominance and under-dominance expression patterns, respectively, were determined if gene expression in the Cross was significantly (*q*-value < 0.05; logFC ≥ 1) higher than that in the high parent or lower than that in the low parent. For genes showing a dominance expression pattern, we used two classification systems to further indicate: (1) which parental line expression phenotype is dominant in the F1, i.e., Leghorn or Fayoumi dominance (2) whether the gene expression in the F1 was elevated to that of the high parent (enhancing dominance) or decreased to that of the low parent (suppressing dominance) (Figure 1).

Average rLog(count) was calculated for each group for the clustering analysis. The DE genes in the brain and liver gene lists were separately analyzed and clustered into 12 clusters using K-means clustering. The optimal numbers of clusters were estimated using a correlation-based Figure of Merit method implemented in SC^2^ATmd tool [25]. K-means clustering was performed in R [26] using Pearson’s correlation method of “amap” package [27]. The percentage of the genes showing non-additive expression patterns was used as a criterion to determine the predominant gene expression patterns in each cluster. When the percentage was greater than 50%, the predominant gene expression pattern of this cluster was considered as non-additive and, if otherwise, additive. The DE genes of each gene expression pattern were further analyzed using DAVID Bioinformatics Resources 6.8 [28]. A threshold Benjamini adjusted *p*-values of 0.05 and an enrichment score greater than 1.3 were applied to identify the gene annotation (GO) terms and KEGG pathways that are significantly enriched by genes in our gene lists. χ^2^ test of independence between ASE and DE genes were conducted in R [26].

## 3. Results and Discussion

### 3.1. Phenotypic Data

**Figure 2:**
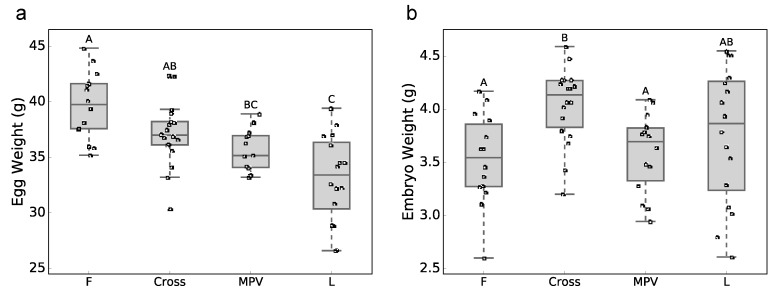
Phenotypic data at embryonic day 12: (**a**) egg weight. (**b**) embryo weight. Means not sharing any letter are significantly different (Tukey-Kramer HSD, *p* < 0.05) (F: Inbred Fayoumi line; L: Inbred Leghorn line; Cross: Fayoumi × Leghorn cross and Leghorn × Fayoumi cross; MPV: mid-parent values).

The mean egg weights for the F (*n* = 14), Cross (FL and LF; *n* = 18) and L (*n* = 16) groups at day 12 of incubation were 39.7 g, 37.1 g, and 33.1 g (Figure 2a), with the Cross and F group significantly higher than that of the L group. The difference in egg weights might be attributed to hen’s age or body weight. The mean of embryo weights for the F, Cross and L groups were 3.53, 4.03 and 3.75 g (Figure 2b). The mean embryo weight of the Cross group was significantly higher than that of the MPV (*p* = 0.0404) and F (*p* = 0.0165) groups, but not significantly different from the L group. Additionally, the Cross group had higher embryonic efficiency than the F group (Figure S1). In summary, the Cross group showed non-additive heterosis effect for embryo weight.

### 3.2. Differentially Expressed Genes between Inbred Fayoumi and Leghorn Lines

**Figure 3:**
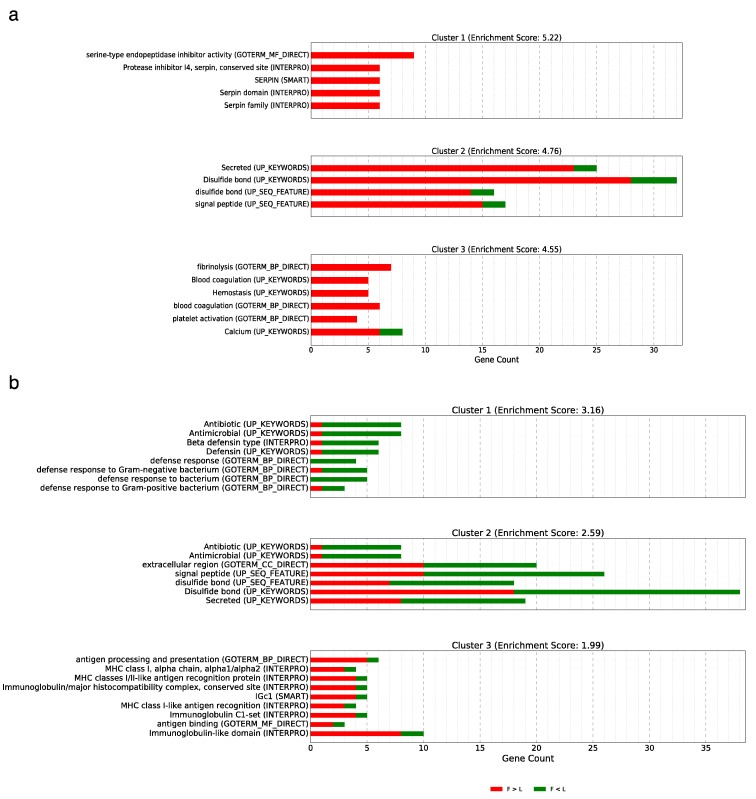
Top 3 functional annotation clusters of the differentially expressed (DE) genes between inbred Fayoumi line (F) and inbred Leghorn line (L) in brain (**a**) and liver (**b**).

Fayoumi and Leghorn chickens are genetically distant and the genetic difference between the two lines is expected to be more pronounced than that between the cross and each of the parental line or between the reciprocal crosses. Here, we compared the difference of gene expression between the two lines at embryonic day 12. This analysis revealed 304 DE genes in the brain and 579 DE genes in the liver, which are the largest number of DE genes among all pairwise analyses in the current study (Table 1) and overlap with a large number of DE genes identified among all other comparisons (Figure S2). Among the DE genes in the brain, 197 and 107 genes were expressed higher, respectively, in Fayoumi and in Leghorn when these two inbred lines were compared one with the other. The top three functional annotation clusters are shown in Figure 3a, with most genes expressed higher in Fayoumi. The genes in cluster 1 (*SPIA1*, *SPIA4*, *SPIA5*, *SPIK5*, *ITIH2*, *ITIH3*, *SERPINC1*, *SERPIND1*, and *AMBP*) are related to serine proteinase and peptidase inhibitor activity, and the genes in both cluster 2 and cluster 3 are mainly involved in blood coagulation and immune-related functions. The DE genes in the brain are also enriched in 13 GO terms and 1 KEGG pathway—metabolic pathway (Table S1). The most attention-drawing biological processes are coagulation and fibrinolysis, which play a central role in brain homeostasis and development [29]. For example, inhibitors of plasminogen activator/plasmin system, such as serine proteinase inhibitor, may block or slow neuronal migration [30,31,32]—a pivotal event in brain development. Both *AvBD9* (ENSGALG00000019845) and *AvBD10* (ENSGALG00000016667) were expressed higher in F than L, implying stronger antimicrobial ability of Fayoumi chickens. Additionally, 45 DE genes were enriched for the GO term extracellular exosome. Extracellular exosome vesicles (EV) mediate intercellular interaction in the nervous system and thereby impacts brain development (reviewed in [33]). The DE genes between Fayoumi and Leghorn suggest a line difference during brain development at embryonic day 12.

The DE genes in the liver include 277 genes expressed higher in Fayoumi and 302 genes expressed higher in Leghorn. Some of the DE genes were enriched in immune-related functions, such as antimicrobial function, defense response, antigen processing and presentation (Figure 3b). The genes encoding for avian β-defensins (*AvBD1*, *AvBD2*, *AvBD6*, *AvBD8*), and genes encoding for Cathelicidin (*CATH3* and *CAMP*) were all expressed at a lower level in Fayoumi than Leghorn. Only *AvBD10* was expressed higher in Fayoumi. Compared with *AvBD10*, the expression levels of *AvBD1, 2, 6, 8* were relatively low, for liver is known to be the main site for *AvBD10* expression but not for the other 4 genes [34,35]. Synthetic AvBD10 has shown a strong inhibition effect on bacteria and fungi in vitro [36], however, there is a lack of study on how liver-expressed β-defensins contribute to innate immunity. It is reasonable to speculate those peptides may exert antimicrobial functions both locally and distantly through blood circulation.

Genes involved in antigen processing and presentation (Figure 3b), such as *BLB2*, *BFIV21*, *YF6*, *MHCIA7*, were expressed at higher levels in Fayoumi chickens than that of Leghorn chickens. Major histocompatibility complex (MHC) proteins play a central role in adaptive immunity. Because the present study doesn’t include pathogen challenge, the DE genes involved in both antimicrobial peptides and antigen processing and presentation demonstrate a basal difference of those genes between the two lines. It has been shown that variant frequency at the MHC locus is different between Fayoumi and Leghorn [37]. Further, expression of MHC Class Iα in spleen, liver, heart, thymus, and bursa was higher in Marek’s disease (MD) resistant haplotype than MD-susceptible haplotype [38]. Interestingly, in the chicken spleen, the highly expressed MHC class I proteins on the cell surface are more specific in peptide binding than the lowly expressed ones [39]. Thus, higher gene expression levels of MHC genes in Fayoumi embryos may suggest superior disease resistance and the specificity of antigen recognition than Leghorn embryos. This corroborates with the previous observation that Fayoumi embryos were found to have a higher survival rate than Leghorn embryos following Rous Sarcoma virus infection [16]. It is also important to note that liver is an immune privileged organ that is inclined to antigen tolerance rather than removal [40], so additional study in other organs of the immune system would be helpful to understand the difference of pathogen resistance between the two lines at the embryonic stage.

### 3.3. Gene Expression of the Reciprocal Crosses

At embryonic day 12, the reciprocal crosses showed generally similar gene expression, as only five DE genes in the brain and six DE genes in the liver were identified. The five DE genes in the brain (ENSGALG00000042468, ENSGALG00000046132, ENSGALG00000044374, ENSGALG00000015374, ENSGALG00000036759) are all encoding for long noncoding RNAs (lncRNA), whose roles are unknown. The DE genes in the liver include *DOCK3*, *LRRK1*, *KLF5*, *HBE*, *PODN*, and *Mt_tRNA*. Although the gene expression profiles were largely similar between FL and LF, more differences in gene expression may emerge in later developmental stages, which could contribute to the reciprocal cross effects for traits measured post-hatch.

### 3.4. Comparison of Gene Expression Pattern across Parental Lines and F1 Crosses

**Figure 4:**
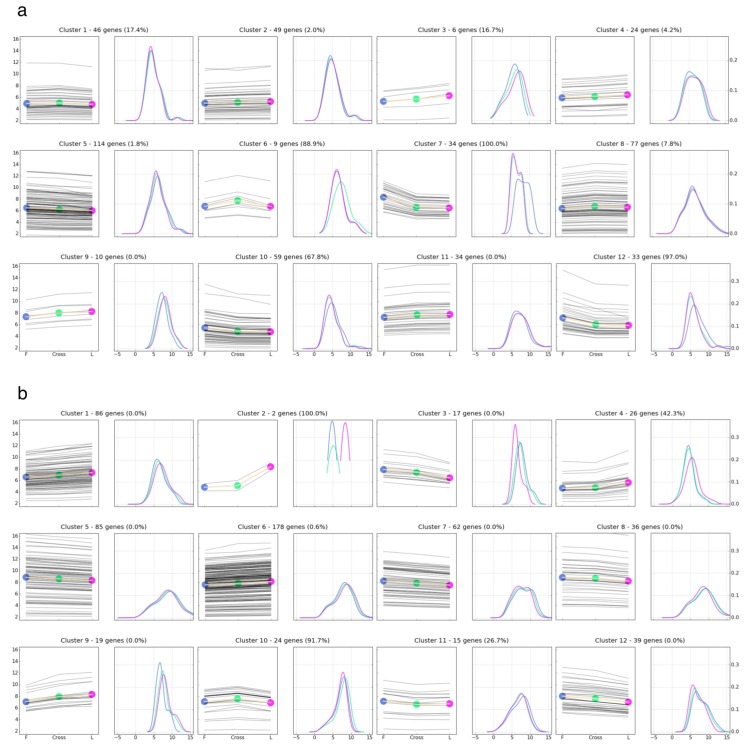
K-means clustering of differentially expressed (DE) genes in the brain (**a**) and in the liver (**b**). For each cluster, the plot on the left shows the average gene expression levels for Fayoumi (F), Cross, and Leghorn (L) groups in rLog(count); the plot on the right shows the kernel density estimation of the three groups; percentages of the genes with non-additive expression pattern were given in parentheses (F: Blue; Cross: Green; L: Fuchsia).

Because only a few genes were differentially expressed between the reciprocal crosses, FL and LF samples were combined as the Cross group, and the DE genes between Cross and parental lines were identified to study gene expression heterosis (Table 1). The DE genes, including F vs. L, F vs. Cross, L vs. Cross, and MPV vs. Cross were clustered into 12 K-means clusters, separately for each tissue type (Figure 4, Figure S3). Overlapping of the DE genes is shown as Venn diagrams in Figure S2. There were 133 genes in the brain and 40 genes in the liver expressed differentially between the Cross and MPV groups. Those genes, which comprised 26.9% and 6.8% of the DE genes in our gene lists from embryonic brain and liver, were considered as showing non-additive transgenerational gene expression pattern, which led to the inference that the majority of the DE genes in our gene lists exhibit an additive expression pattern. As the percentages of genes with non-additive expression patterns were indicated in parentheses for each K-means cluster (Figure 4), the main gene expression pattern for clusters 6, 7, 10 and 12 in the brain and clusters 2 and 10 in the liver were non-additive, and additive for the remaining clusters. Genes showing non-additive expression patterns were further categorized into dominance, over-dominance and under dominance expression patterns. As a result, there were five genes in the brain and two genes in the liver showing over-dominance expression pattern, and 128 genes in the brain and 38 genes in the liver showing dominance expression pattern. Of genes showing dominance expression pattern, most were of Leghorn dominance in the brain and of Fayoumi dominance in the liver. No genes showed the under-dominance pattern with statistical significance. Overall, our result revealed additivity as the predominant transgenerational gene expression pattern between the F1 cross and parental lines. Dominance and over-dominance gene expression patterns were second and third in frequency, respectively.

Transgenerational gene expression pattern and gene expression heterosis have been examined in other species. All gene expression patterns were previously reported between maize F1 hybrids and their parents, with additivity as the main pattern (78%) and dominance as the second main pattern [10]. A study in rice suggested quantitative trait loci (QTLs) of additive effect comprised 50%, while QTLs of dominance and over-dominance effect separately comprised about 30% [41]. Rapp et al. found most genes were expressed in an additive pattern in allopolyploid cotton [42]. Out of those genes with additive pattern, Rapp et al. also defined “expression dominance” to describe the pattern that gene expression in hybrids is not significantly different from one of the parental lines and MPV, but gene expression of this parental line, allopolyploid and MPV is required to be all significantly different from the other parental line. As a result, and they found “expression dominance” is the most prevalent pattern [42]. Genes falling into “expression dominance” are classified as additivity in our study.

### 3.5. Implications and Functional Annotation of the Differentially Expressed Genes Showing a Non-Additive or Additive Expression Pattern

#### 3.5.1. Over-Dominance

There were five genes expressed in an over-dominance pattern in the brain, including two novel genes (ENSGALG00000042217 and ENSGALG00000031253), a gene encoding lincRNA (ENSGALG00000043625), a gene encoding Mt_tRNA (ENSGALG00000033462), and a gene encoding lactate dehydrogenase D (*LDHD*, ENSGALG00000023828).

Both Mt_tRNA and lactate dehydrogenase D are involved in protein synthesis in the mitochondrion and thereby potentially affect energy production. Polyadenylation of tRNA after transcription is critical for its stability and maturation [43,44]. It is also essential for the degradation of incorrectly folded tRNA in bacteria [45], hypomodified initiator tRNA in yeast [46,47], and tRNA in human mitochondria [48]. Therefore, high polyadenylation may suggest a high turnover rate of this tRNA. The over-dominance pattern of this *Mt_tRNA* gene could result from either overall high gene transcription or high polyadenylation ratio of transcripts in the Cross group. Additionally, polyadenylation was found to function as a discriminator for two tRNAs that are encoded by overlapping mitochondrial tRNA genes in chickens [49]. Despite the cause being uncertain, the results suggest that the Cross chickens have elevated protein synthesis activity compared to both parental lines in brain mitochondria.

*LDHD* is expressed at a relatively low level in the brain (FPKM < 2 in the Cross and < 1 in F and L), which could be related to the low abundance of D-lactate [50]. D-lactate could interfere with biological processes that use L-lactate as a substrate, like TAC cycle, and impair respiration efficiency in mitochondria [51]. Higher expression of LDHD in the Cross group implies a more efficient energy production in crosses compared with parental lines. In agreement with our inference, *LDHD* expression was previously found up-regulated in breast skeletal muscle of high feed efficiency broiler chickens [52].

There were two genes of over-dominance pattern in the liver: *RN7SL1*(ENSGALG00000026904) and a novel gene (ENSGALG00000040994). *RN7SL1, or Metazoa_SRP*, is cytosolic lncRNA that mediates translocation of secretory protein across the endoplasmic reticulum membrane [53]. It was also found present in tumor exosomes [54,55,56]. Taken together, the over-dominance genes in the brain indicated a higher mitochondrial activity in the crossbreds, yet the consequence caused by over-dominance genes in the liver remains unclear.

#### 3.5.2. Dominance

In the brain, 128 genes showed the dominance expression pattern. Gene expression of 13 DE genes in Cross were similar to Fayoumi but significantly different from Leghorn (designated as the Fayoumi dominance pattern), while 115 genes were similar to Leghorn but significantly different from Fayoumi (designated as Leghorn dominance pattern). The genes of the Fayoumi dominance pattern were five novel genes (ENSGALG00000003333, ENSGALG00000037297, ENSGALG00000040379, ENSGALG00000041375, ENSGALG00000046355), *OBSL1* (ENSGALG00000011242), *REM1* (ENSGALG00000045602), *TPBGL* (ENSGALG00000030652), *ATP2C2* (ENSGALG00000035743), *ATP10B* (ENSGALG00000001662), *CHIR-IG1-5* (ENSGALG00000029472), a gene encoding for lectin-like type II transmembrane protein (ENSGALG00000033116) and a gene encoding for C-type lectin domain family 2 member D-like (ENSGALG00000033672). OBSL1 is a cytoskeletal adapter protein [57] and plays a vital role in dendrites morphogenesis [58]. REM1 is a GTPase [59] and potentially involved in cytoskeletal changes that affect neuron morphology and migration [60]. Both genes were expressed higher in F and Cross than in L, i.e. the gene expression in Cross elevates to the level of the high parent for these genes (F). We refer to this expression pattern as “enhancing dominance”, and “suppressing dominance” if the gene expression in Cross decreases to the level of the low parent for the gene. In fact, 10 out of 13 genes showing the Fayoumi dominance pattern were of the enhancing dominance. Only *ATP2C2*, *ATP10B* and a novel gene (ENSGALG00000046355) were showing the suppressing dominance pattern. ATP2C2, ATP10B, CHIR-IG1-5, and lectin-like type II transmembrane protein are all transmembrane proteins (enrichment score 1.54), but *CHIR-IG1-5* and gene for lectin-like type II transmembrane protein were showing the enhancing dominance pattern.

The majority of the DE genes with a non-additive expression pattern in the brain showed the Leghorn dominance pattern. Contrary to the genes of Fayoumi dominance pattern, most (108 out of 135) genes with the Leghorn dominance expression pattern were showing suppressing dominance. For example, defensin genes *AvBD9* and *AvBD10* were showing the suppressing dominance pattern. Further functional analysis suggested the genes of Leghorn dominance pattern are enriched in functions and biological processes similar to that of DE genes between F and L (Table S2), such as coagulation and fibrinolysis. In addition, the genes of Leghorn dominance pattern were enriched in KEGG pathways such as Metabolic pathways, Tyrosine metabolism, Biosynthesis of amino acids (Table S2). These results suggest that, in the embryonic brain, the activity of these metabolic pathways is similar in the F1 crosses and Leghorn, and possibly lower than that in Fayoumi.

In the liver, 38 genes were expressed in dominance pattern, 31 genes of Fayoumi dominance pattern and seven genes of Leghorn dominance pattern. There were 17 genes of Fayoumi dominance showing the enhancing dominance pattern, 16 of which, however, are novel genes. The remaining gene (ENSGALG00000029094) encodes for lncRNA RN7SL1. The genes of suppressing dominance pattern were enriched in keywords such as defensin, antibiotic and antimicrobial in DAVID analysis, because of differential expression of *AvBD1*, *2*, *6*, and *CATH2* between Leghorn and Cross. The genes of Leghorn dominance pattern included three genes of enhancing dominance and four genes of suppressing dominance. *DHCR24* and *SQLE* are enriched in Steroid biosynthesis pathway (Table S2), and both are of suppressing dominance.

The DE genes encoding for antimicrobial peptides in both brain and liver were all expressed in suppressing dominance pattern, which may provide some clues to understand the previous observation that Fayoumi and Leghorn crosses and Leghorn pure line showed similar resistance to Rous sarcoma virus [16,61]. Additionally, most genes involved in metabolic pathways in both tissues also showed suppressing dominance with Leghorn dominance pattern. As embryonic weight and embryonic efficiency were found greater in the Cross and Leghorn than in Fayoumi, it is possible that in the F1 embryos, Leghorn-derived regulatory elements are more influential in regulating expression of genes involved in metabolism and modulate embryonic development and growth. However, additional examination of other metabolic organs and tissues is necessary to fully understand the molecular mechanisms responsible for this difference.

#### 3.5.3. Additive

There were 257 DE genes in the brain in this category (Figure S2), but no GO terms or pathway were enriched with statistical significance in DAVID analysis. Most of the genes encode for transmembrane proteins (enrichment score 2.06), and are involved a variety of processes, such as genes encoding for ATPase (*ATP6*, *ATP8B1*), antigen presenting protein (*BF2*, *BLB2*), Cytochromes P450 enzyme (*CYP1A1*, *CYP2C23*, *CYP4V2*), and receptors for growth hormone (*GHRZ*), glycine (*GLRA4*), interleukin 13 (*IL13RA2*), thyroid stimulating hormone (*TSHR*) etc.

In the liver, 197 genes belonging to this category were enriched in GO terms and pathways such as antigen processing and presentation, steroid hormone biosynthesis, and metabolic pathways (Figure S2, Table S2). Those pathways were also enriched in the differential gene expression analysis between F and L. The genes involved in antigen processing and presentation (*YF2*, *BF2*, *BLB2*, *MHCIA7*) were expressed significantly higher in Cross than in L, but there was no significant difference in gene expression between Cross and F, a similar pattern observed for *BF2* and *BLB2* expression in the brain. These results may indicate that the ability of antigen presenting and processing in the crosses is superior to Leghorn to some extent, but it is still not comparable to Fayoumi. About half of the DE genes enriched in steroid hormone synthesis and metabolic pathways were DE genes in the F vs. Cross analysis while the other half were identified in the L vs. Cross analysis. Despite the epistatic relationship not being directly evaluated here, some of those DE genes could potentially be involved in epistatic interactions with other genes and contribute to heterosis.

### 3.6. Cis- vs. Trans-Regulation of Gene Expression

Understanding how gene expression is regulated is important to uncover mechanisms underlying the differential expression of genes between parental lines and between the crosses and the parental lines and, subsequently, the molecular mechanism of heterosis. It was reported both *cis*- and *trans*-regulatory mechanisms contribute to heterosis in plants [62,63]. Another study in rice found more than 40% of DE genes between F1 and parents were potentially caused by ASE [64], suggesting that differential expression in these genes are driven by *cis*-acting regulatory elements. We previously identified 2242 ASE genes in the brain and 1735 ASE genes in the liver in reciprocal crosses [18]. After excluding the ASE genes with multiple SNPs that showed conflicting preferred alleles, we checked the genes that were identified as both ASE and DE. Less than 10% of DE genes in the brain and less than 20% of DE genes in the liver were identified as both ASE and DE, most of which showed additive gene expression pattern (Table 2). For the majority of DE genes between parental lines and between crosses and parental lines, gene expression of the two parental alleles didn’t show a significant difference in the F1 crosses. These results suggest that the *cis*-acting regulation of gene expression is not the main mechanism leading to differential expression between parental lines and between the crosses and parental lines. Therefore, *trans*-acting regulation of gene expression may be largely responsible for differential gene expression and play more important roles in heterosis.

## 4. Conclusions

To the best of our knowledge, this is the first transcriptomic study to investigate transgenerational gene expression patterns, i.e., gene expression inheritance, in chickens. Our phenotypic data suggested heterosis effect in embryo weight. When comparing gene expression between F1 crosses and the inbred parental lines, we found additivity was the predominant gene expression pattern and dominance and over-dominance were the second and third, respectively. The DE genes between parental lines and between the crosses and the parental lines were enriched in functions and pathways related to tissue development and immunity in embryonic brain, and immunity and metabolism in embryonic liver. We also found most DE genes did not exhibit allelic imbalance in gene expression in the crosses, which suggested *trans*-acting mechanisms may be the main regulatory mechanisms underlying differential gene expression between parental lines and heterosis in the F1 cross. This study focused on gene expression analysis in brain and liver at an early development stage (embryonic day 12), and thus the DE genes and observed gene expression patterns may only partly explain the phenotypic heterosis effect in the F1 cross. As gene expression is tissue and developmental stage dependent, future research of gene expression in other tissues and development stages would provide additional insights into heterosis in chickens.

## Figures and Tables

**Table 1:** Number of differentially expressed genes.

	Brain	Liver
F vs. L	304	579
F vs. Cross	310	91
L vs. Cross	73	147
FL vs. LF	5	6
MPV vs. Cross	133	40

Abbreviations: F: Inbred Fayoumi line; L: Inbred Leghorn line; FL: Fayoumi × Leghorn cross; LF: Leghorn × Fayoumi cross; Cross: FL and LF; MPV: mid-parent gene expression values.

**Table 2:** Overlapping matrix between genes showing differential expression and allele-specific expression.

	A	OD	D_F	D_L	ASE_F	ASE_L
Brain						
A	362	-	-	-	-	-
OD	0	5	-	-	-	-
D_F	0	0	13	-	-	-
D_L	0	0	0	115	-	-
ASE_F	16	0	0	2	674	-
ASE_L	15	0	0	1	0	736
Liver						
A	549	-	-	-	-	-
OD	0	2	-	-	-	-
D_F	0	0	31	-	-	-
D_L	0	0	0	7	-	-
ASE_F	62*	0	0	1	632	-
ASE_L	45*	0	0	0	0	608

Abbreviations: A: Additive; OD: Over-dominance; D_F: Fayoumi dominance; D_L: Leghorn dominance; ASE: allele-specific expression; ASE_F: ASE genes with Fayoumi allele expressed higher in the F1 cross; ASE_L: ASE genes with Leghorn allele expressed higher in the F1 cross; DE: differential expression; DE_F: DE genes between F and L with expression in F greater than expression in L; DE_L: DE genes between F and L with expression in L greater than expression in F. *significant (*p* < 0.05, χ^2^ test ) overlap between DE and ASE genes.

## Supplementary Materials

The following are available online at http://www.mdpi.com/2073-4425/10/1/27/s1, Figure S1: Embryo efficiency for parental line and crosses, Figure S2: Venn diagram of DE genes, Figure S3: Optimal cluster number estimation, Table S1: GO terms and pathway enrichment for DE genes between F and L, Table S2: Functional analysis of the genes showing dominance and additive expression patterns in the embryonic brain and liver.
