# Supplementary material for: RNA-Seq Analyses Identify Additivity as the Predominant Gene Expression Pattern in F1 Chicken Embryonic Brain and Liver
Source: Genes (Basel). 2019 Jan 7;10(1):27. doi: 10.3390/genes10010027 (PMC6356826; doi:10.3390/genes10010027)
Supplement: Supplementary file 1 [file genes-10-00027-s001.pdf]

genes

Supplementary File

RNA-Seq Analyses Identify Additivity as the Predominant Gene Expression Pattern in F1 Chicken Embryonic Brain and Liver

Zhu Zhuo [1], Susan J. Lamont [2] and Behnam Abasht [1,*]

[1] Department of Animal and Food Sciences, University of Delaware
[2] Department of Animal Science, Iowa State University
* Correspondence: abasht@udel.edu; Tel.: +1-302-831-8876

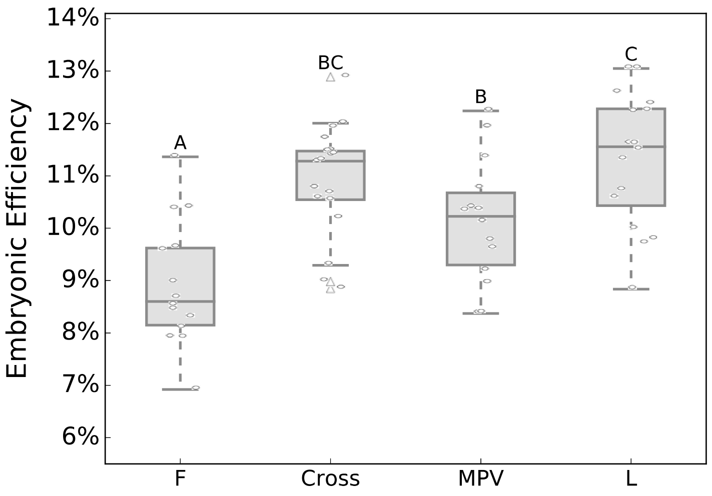

Figure S1. Embryo efficiency for parental line and crosses.

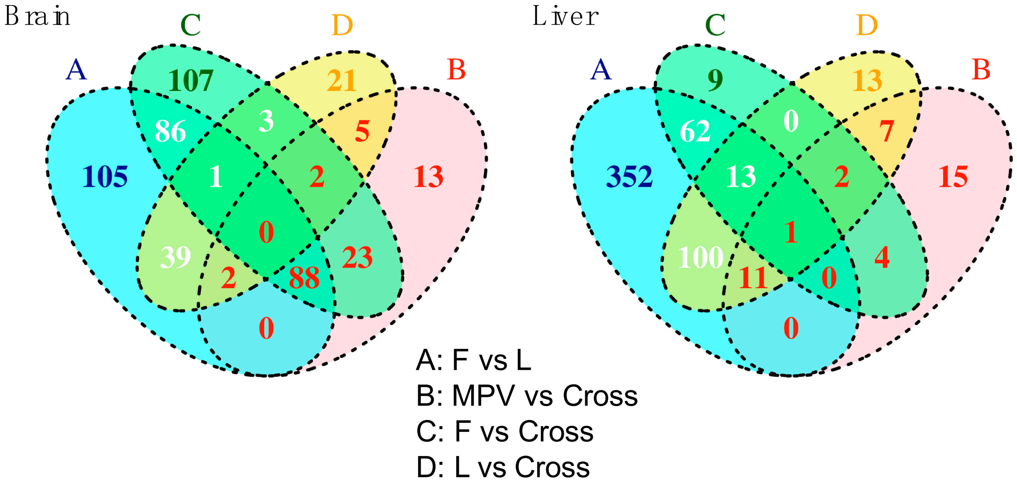

Figure S2. Venn diagram of DE genes.

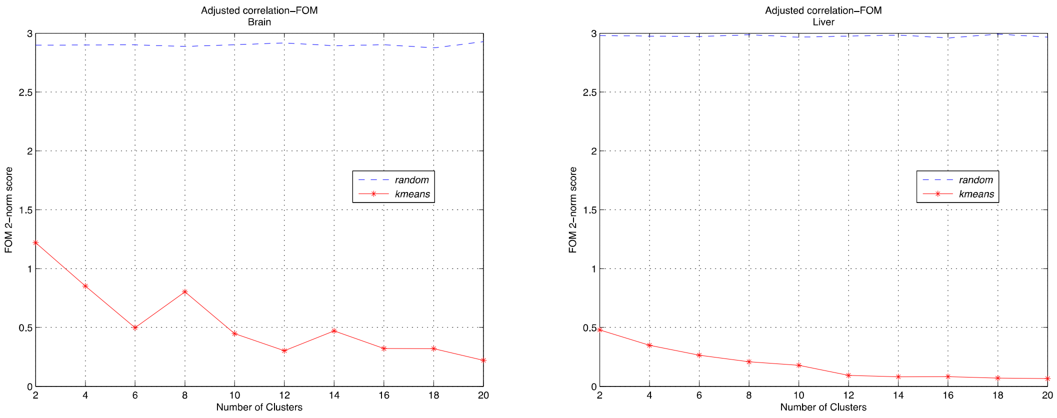

Figure S3. Optimal cluster number estimation.

Table S1. GO terms and pathway enrichment for DE genes between F and L.

Caterogy	Term	Gene Count	Fold Enrichment	FDR
Brain				
GOTERM_BP_DIRECT	GO:0042730~fibrinolysis	7	47.34	8.34E-07
GOTERM_BP_DIRECT	GO:0031639~plasminogen activation	4	45.08	1.39E-02
GOTERM_BP_DIRECT	GO:0007596~blood coagulation	6	11.27	2.54E-02
GOTERM_CC_DIRECT	GO:0072562~blood microparticle	15	22.35	1.44E-13
GOTERM_CC_DIRECT	GO:0005576~extracellular region	19	4.63	5.89E-06
GOTERM_CC_DIRECT	GO:0070062~extracellular exosome	45	2.08	4.39E-05
GOTERM_CC_DIRECT	GO:0005615~extracellular space	22	2.51	3.63E-03
GOTERM_MF_DIRECT	GO:0004867~serine-type endopeptidase inhibitor activity	9	13.43	5.10E-05
GOTERM_MF_DIRECT	GO:0008392~arachidonic acid epoxygenase activity	4	64.15	1.50E-03
GOTERM_MF_DIRECT	GO:0004252~serine-type endopeptidase activity	9	7.70	1.31E-03
GOTERM_MF_DIRECT	GO:0008395~steroid hydroxylase activity	4	36.66	6.31E-03
GOTERM_MF_DIRECT	GO:0020037~heme binding	8	6.26	1.09E-02
GOTERM_MF_DIRECT	GO:0005506~iron ion binding	8	5.40	2.22E-02
KEGG_PATHWAY	gga01100:Metabolic pathways	27	1.78	4.33E-02
Liver				
GOTERM_CC_DIRECT	GO:0030141~secretory granule	8	8.16	9.66E-03
KEGG_PATHWAY	gga00140:Steroid hormone biosynthesis	8	6.83	1.35E-02
KEGG_PATHWAY	gga01100:Metabolic pathways	56	1.53	2.19E-02

Table S2. Functional analysis of the genes showing dominance and additive expression patterns in the embryonic brain and liver.

Caterogy	Term	Gene Count	Fold Enrichment	FDR
Genes of Leghorn Dominance in brain				
GOTERM_BP_DIRECT	GO:0042730~fibrinolysis	7	91.24	8.81E-09
GOTERM_BP_DIRECT	GO:0007596~blood coagulation	6	21.72	9.63E-04
GOTERM_BP_DIRECT	GO:0031639~plasminogen activation	4	86.90	7.81E-04
GOTERM_BP_DIRECT	GO:0042632~cholesterol homeostasis	5	17.61	1.17E-02
GOTERM_BP_DIRECT	GO:0034116~positive regulation of heterotypic cell-cell adhesion	3	130.35	9.64E-03
GOTERM_BP_DIRECT	GO:0030195~negative regulation of blood coagulation	3	78.21	2.63E-02
GOTERM_BP_DIRECT	GO:0051258~protein polymerization	3	78.21	2.63E-02
GOTERM_BP_DIRECT	GO:0010873~positive regulation of cholesterol esterification	3	78.21	2.63E-02
GOTERM_BP_DIRECT	GO:0051006~positive regulation of lipoprotein lipase activity	3	65.17	3.35E-02
GOTERM_CC_DIRECT	GO:0072562~blood microparticle	16	44.15	1.30E-19
GOTERM_CC_DIRECT	GO:0070062~extracellular exosome	37	3.17	1.08E-09
GOTERM_CC_DIRECT	GO:0005576~extracellular region	14	6.32	4.19E-06
GOTERM_CC_DIRECT	GO:0005615~extracellular space	19	4.02	8.04E-06
GOTERM_CC_DIRECT	GO:0042627~chylomicron	4	70.95	1.89E-04
GOTERM_CC_DIRECT	GO:0034361~very-low-density lipoprotein particle	4	49.66	5.31E-04
GOTERM_CC_DIRECT	GO:0005577~fibrinogen complex	3	62.08	7.45E-03
GOTERM_CC_DIRECT	GO:0034364~high-density lipoprotein particle	3	53.21	9.08E-03
GOTERM_CC_DIRECT	GO:0031091~platelet alpha granule	3	37.25	1.70E-02
GOTERM_CC_DIRECT	GO:0005623~cell	6	5.17	3.21E-02
GOTERM_MF_DIRECT	GO:0004867~serine-type endopeptidase inhibitor activity	8	19.60	1.52E-05
GOTERM_MF_DIRECT	GO:0004252~serine-type endopeptidase activity	8	11.24	3.69E-04
KEGG_PATHWAY	gga01100:Metabolic pathways	24	2.45	2.12E-04
KEGG_PATHWAY	gga00980:Metabolism of xenobiotics by cytochrome P450	6	21.06	1.62E-04
KEGG_PATHWAY	gga00350:Tyrosine metabolism	5	17.55	2.47E-03
KEGG_PATHWAY	gga00982:Drug metabolism - cytochrome P450	4	15.55	2.35E-02

Genes of Fayoumi Domiance in Liver				
GOTERM_BP_DIRECT	GO:0042742~defense response to bacterium	3	81.76	1.83E-02
GOTERM_CC_DIRECT	GO:0005576~extracellular region	4	12.19	4.44E-02
Genes of Leghorn Domiance in Liver				
KEGG_PATHWAY	gga00100:Steroid biosynthesis	2	256.06	1.17E-02
Additive genes in Liver				
GOTERM_BP_DIRECT	GO:0019882~antigen processing and presentation	5	19.12	4.04E-02
GOTERM_BP_DIRECT	GO:0090286~cytoskeletal anchoring at nuclear membrane	4	36.71	2.46E-02
GOTERM_CC_DIRECT	GO:0030141~secretory granule	5	16.51	2.10E-02
KEGG_PATHWAY	gga00140:Steroid hormone biosynthesis	6	14.77	3.03E-03
KEGG_PATHWAY	gga00100:Steroid biosynthesis	4	19.70	3.52E-02
KEGG_PATHWAY	gga01100:Metabolic pathways	24	1.89	2.48E-02
